# Giant sigmoid diverticulum

**DOI:** 10.11604/pamj.2014.17.183.3786

**Published:** 2014-03-10

**Authors:** Olaoye Iyiade Olatunde

**Affiliations:** 1 University of Ilorin Teaching Hospital, Ilorin, Nigeria

**Keywords:** Giant, sigmoid, diverticulum

## Abstract

Giant sigmoid diverticuli are uncommon and usually seen in association with multiple colonic diverticuli. A solitary diverticulum in an otherwise normal colon is very rare. Mostly asymptomatic, excision of giant sigmoid diverticuli is advised to prevent complications.

## Introduction

Giant sigmoid diverticuli are rare. They are commonly asymptomatic and accidentally discovered on abdominal examination. They can however become infected, they can perforate and sometimes fistulate into adjacent organs. Excision is advised to prevent complications.

## Patient and observation

**Figure 1:**
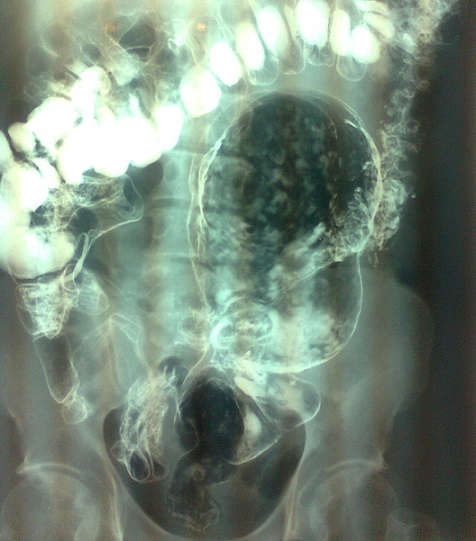
Double contrast barium enema showing a bilobed giant sigmoid diverticulum

A 65 year old man presented with a left lower abdominal mass of two years duration. His bowel habits were normal. He had an uneventful trans-vescical prostatectomy about 8 years prior to presentation. Examination revealed a non tender mass in the left lower abdomen. Mass was more mobile in the transverse plane and tympanitic. Rectal examination was normal. Double contrast barium enema (Figure 1) and CT scan suggested a giant sigmoid diverticulum. Laparotomy revealed a bi-lobed giant diverticulum located in the lower sigmoid colon. It was inseparably adhered (but not communicating) to the medial side of the caecum and to the superior surface of the urinary bladder. There were few discrete paracolic nodes adjacent to the diverticulum. The proximal sigmoid colon and the rectum appeared normal. Diverticulectomy with a Hartmann's procedure was done. Dense adhesions between the diverticulum and the caecum necessitated a limited right hemi colectomy with ileo-colic anastomosis. Recovery was uneventful and he was discharged ten days post operation. Histology revealed no features of malignancy.

## Discussion

Giant sigmoid diverticula are rare with less than 150 cases described in the literature [1]. First described in 1946, they are complications of colonic diverticulosis and are usually seen in elderly patients (average age 65 years) [2]. Colonic diverticulosis is relatively uncommon in tropical Africa because of a predominantly high fibre diet [3]. The presence of a solitary giant sigmoid diverticulum in an otherwise normal colon as seen in this patient is particularly rare.

Most giant sigmoid diverticuli are discovered at routine physical examination but some present with unspecific gastroenterological symptoms. Complications include inflammation, perforation, fistulation into the genito-urinary tract, obstruction and volvulus. Adenocarcinoma has been reported adjacent to a giant diverticulum [4]. This patient had dense adhesions but no fistulae into the bladder. Barium enema, CT scan and MRI are usually diagnostic. Colonoscopy is not. Early segmental resection of giant diverticuli (one or two stage procedure) is recommended to prevent complications [5]. The presence of dense adhesions and a suspicion of coexisting malignancy may necessitate a wider local resection. A right hemi colectomy was necessitated in our patient because of dense adhesions to the caecum and the possibility of malignancy.

## Conclusion

Giant sigmoid diverticuli are rare and usually part of colonic diverticulosis. A solitary diverticulum in an otherwise normal colon is uncommon. Commonly asymptomatic, serious complications can be avoided by early segmental resection of the diverticulum.
